# Editorial: Understanding the Interaction Between Physical Activity and Diet for the Promotion of Health and Fitness

**DOI:** 10.3389/fnut.2021.835535

**Published:** 2022-01-13

**Authors:** Karsten Koehler, Clemens Drenowatz

**Affiliations:** ^1^Department of Sport and Health Sciences, Technical University of Munich, Munich, Germany; ^2^Division of Sport, Physical Activity and Health, University of Education Upper Austria, Linz, Austria

**Keywords:** exercise, eating behavior, weight loss, lifestyle, long-term health, energy balance

## Introduction

The benefits of regular physical activity and a healthy diet are well-documented in the literature (1, 2). However, efforts to counter the global obesity epidemic and related metabolic diseases through interventions focusing on either physical activity or diet alone have been of limited success (3, 4), highlighting the need for new, more refined and integrated approaches. Although it appears intuitive that interventions combining both lifestyle components have the capacity to result in greater health benefits than singular approaches (5), it is also possible that changing behavior related to one component may result in compensatory changes in the other. For example, the obvious health benefits of increased physical activity may be overridden when the resulting increase in energy expenditure is compensated by an increase in dietary energy intake and unhealthier food choices (6). Likewise, dietary interventions such as caloric restriction may result in compensatory reductions in physical activity behavior (7), which could negatively affect physical fitness and performance, undermine weight loss success, and expose individuals to greater risk for future weight regain and other associated diseases (8–10).

The goal of this Research Topic was to strengthen our understanding of how physical activity and diet are related with each other in the context of health and fitness promotion. As such, this Research Topic includes research targeting physical activity, which refers to any bodily movement that results in energy expenditure, as well as exercise, a subset of physical activity with the goal of maintaining or improving physical fitness (11). The Research Topic combines a total of 9 original studies and systematic reviews, which cover three basic themes ranging from the interplay between nutrition and physical activity for weight loss (theme 1), the impact of exercise on food intake regulation (theme 2) and the potential for negative health consequences of excessive exercise (theme 3).

## Theme 1: Weight Loss and Body Composition

Despite considerable efforts, global rates of overweight, and obesity continue to rise and excess body weight is considered a major threat to future public health (12). Accordingly, various strategies, including attempts to alter diet and physical activity, have been implemented to tackle the obesity epidemic. In their systematic review Correia et al. highlight beneficial effects of intermittent fasting on body weight. Diet-induced changes in body weight, however, are generally short lived and even greater benefits can be accomplished with the inclusion of exercise training. Exercise also plays an important role for maintaining lean body mass during caloric restriction as indicated by Roth et al. who reported a decline in lean mass during caloric restriction despite assuring a high protein intake. In addition to structured programs, lifestyle adjustments also play a critical role in weight loss. Myers et al. show that particularly vigorous physical activity, along with a reduction in energy-dense foods, was associated with a more pronounced weight loss in women. Similarly, van Baak et al. report greater weight loss during caloric restriction in participants who increase their physical activity. Furthermore, increased physical activity was associated with beneficial changes in various cardio-metabolic risk factors and weight loss maintenance beyond the intervention period.

## Theme 2: Exercise and Food Intake Regulation

While physical activity and exercise increase energy expenditure, most exercisers increase their dietary energy intake. This phenomenon, often referred to compensatory eating, was also described in a recent study by Horner et al. who showed that neither gastric emptying nor appetite-regulating hormones were significantly altered after a 4-week exercise intervention, suggesting that short-term changes in gastro-intestinal regulation play no major role in compensatory eating. Post-exercise food intake was also studied by Okada et al. who reported that administration of exogenous ketones impacted appetite-regulating hormones but failed to affect appetite perception and post-exercise energy intake. These two interventions are complemented by a systematic review and meta-analysis by Hubner et al. on the effects of exercise on appetite regulation in older adults. Despite limited research in this demographic, exercise, and physical activity appear to promote satiety sensitivity and appetite control, thereby providing an avenue for reducing disease burden later in life.

## Theme 3: Possible Negative Consequence

Besides the beneficial effects of physical activity and exercise there are also some possible harmful effects that need to be acknowledged. Ribeiro et al. discuss the potential detrimental effects of exacerbated exercise on the gastrointestinal environment that can, among others, impair gastric motility, and nutrient absorption. Moore et al. further address the increased risk for low energy availability in endurance athletes, and emphasize the need for adequate dietary energy intake during periods of high energy demands. The overall benefits of physical activity, however, should not be questioned by these results. Rather, these studies highlight the complex interaction between diet and physical activity and their effects on the human body and health.

## Summary

This overview of current research related to physical activity and diet highlights the importance of integrating both components regardless whether the goal is to maximize weight loss or to diminish the potential negative effects at the upper end of the activity spectrum. A deeper understanding of the interaction between these two critical lifestyle approaches is required for the development of combined interventions involving physical activity and diet that result in successful, long-term health improvements while avoiding unhealthy compensatory behavior in the other domain.

## Author Contributions

KK and CD wrote the introduction and the summary. CD summarized the publications pertaining to weight loss and excessive exercising. KK wrote the summaries of publications relating to the impact of exercise on food intake regulation. Both authors approved the submitted version.

## Conflict of Interest

The authors declare that the research was conducted in the absence of any commercial or financial relationships that could be construed as a potential conflict of interest.

## Publisher's Note

All claims expressed in this article are solely those of the authors and do not necessarily represent those of their affiliated organizations, or those of the publisher, the editors and the reviewers. Any product that may be evaluated in this article, or claim that may be made by its manufacturer, is not guaranteed or endorsed by the publisher.
